# Identification of risk factors for type 2 diabetes across age-based proxies of early and late postmenopausal stages in prediabetic women

**DOI:** 10.3389/fendo.2026.1891487

**Published:** 2026-09-15

**Authors:** Juan Carlos Lizarzaburu-Robles, Ignacio Mahíllo-Fernández, Amalia Paniagua, Maite Ortega Juaristi, Blanca Timón, Sacramento Martínez-Albaladejo, Sebastián Mas-Fontao, Clotilde Vázquez, Oscar Lorenzo

**Affiliations:** 1 PhD Program in Medicine and Surgery, Doctoral School, Autónoma University, Madrid, Spain; 2 Laboratory of Vascular Pathology and Diabetes, Instituto de Investigación Sanitaria (IIS)-Fundación Jiménez Díaz, Autónoma University, Madrid, Spain; 3 Biostatistics and Epidemiology Unit, Instituto de Investigación Sanitaria (IIS)-Fundación Jiménez Díaz, Autónoma University, Madrid, Spain; 4 Endocrinology Department, Fundación Jiménez Díaz University Hospital, Madrid, Spain; 5 Spanish Biomedical Research Centre in Diabetes and Associated Metabolic Disorders (CIBERDEM) Network, Madrid, Spain

**Keywords:** ageing, early post-menopause, late post-menopause, prediabetes, type 2 diabetes

## Abstract

**Background:**

Prediabetes is increasingly prevalent, and women transitioning through menopause may face a particularly elevated risk of progressing to type 2 diabetes (T2DM). Estrogen deficiency promotes adiposity, insulin resistance, and inflammation, potentially amplifying the metabolic burden of aging. However, the determinants of T2DM in these women remain insufficiently characterized.

**Methods:**

This retrospective study included 229 prediabetic women evaluated at a tertiary-care hospital and followed for up to 5.5 years. Prediabetes was defined according to ADA criteria and women were classified as premenopausal (< 51 years), early postmenopausal (51–60 years), or late postmenopausal (> 60 years). Clinical and biochemical variables were analyzed for hazard ratios (HR) of T2DM using Cox regression, and Kaplan-Meier curves.

**Results:**

During follow−up, 18% of premenopausal and 35% of postmenopausal women developed T2DM. Higher BMI predicted T2DM in both groups. In postmenopausal women, IGT (HR: 2.59), hypertension (HR: 3.91), lower HDL−C (HR: 0.96), lower vitamin D (HR: 0.93), and reduced eGFR (HR: 0.97) were independently associated with T2DM. Stratification revealed that early post-menopause carried the highest incidence of T2DM. In early post-menopause, higher BMI and uric acid, and lower vitamin D were independent predictors, whereas in late post-menopause, hypertension and reduced eGFR were the strongest determinants.

**Conclusions:**

Risk Factors associated with progression to T2DM in prediabetic women appear to differ across age-defined menopausal stages. Excess adiposity remains a key factor, whereas early post-menopause is characterized by additional associations with hyperuricemia and vitamin D deficiency. In late post-menopause, hypertension and declining renal function appear to be more prominent. These findings support stage-specific risk assessment and tailored preventive strategies in prediabetic women.

## Introduction

The rising prevalence of prediabetes has increased the number of individuals at risk of developing type 2 diabetes (T2DM) (1). Obesity and aging are major contributors to T2DM, and in women, the postmenopausal decline in estrogen levels further promotes visceral adiposity and insulin resistance (2, 3). Each year, about two million women reach menopause and spend roughly 40% of their lives in the postmenopausal stage (4). Estrogen deficiency affects adipose tissue, liver, skeletal muscle, and vascular endothelium, leading to increased lipogenesis, decreased fatty acid oxidation and insulin sensitivity, and the loss of anti−inflammatory and vasoprotective actions (4, 5). Thus, obesity, aging, and menopause may form a deleterious triad of interconnected factors that substantially heightens susceptibility to T2DM in prediabetic women (6). However, despite this increased cardiometabolic risk, preventive strategies are not personalized and remain similar to those recommended for the general population, including lifestyle interventions and management of cardiometabolic risk factors (i.e., hypertension, hyperlipidemia, disglycemia) (7).

Postmenopausal women with prediabetes may exhibit a distinct cardiometabolic profile driven by estrogen deficiency, visceral adiposity, and age−related metabolic changes (8). Glycemic markers, as well as specific factors related to adiposity, lipogenesis, inflammation, and liver or kidney injury, may be altered in this population. Consequently, the interaction between aging, estrogen deficiency, and metabolic dysregulation may amplify the risk of progression from prediabetes to T2DM (3, 9). However, few epidemiological studies addressed the risk factors across postmenopausal stages (10, 11). Aging further intensifies the metabolic consequences of estrogen deficiency, and according to the STRAW + 10 staging system and cardiovascular epidemiology, age 60 may represents a critical threshold marking the late postmenopausal stage (12). Therefore, understanding how postmenopausal phases and age interact with metabolic parameters could be essential for improving risk assessment and early identification of prediabetic women at risk of T2DM. The aim of this study was to identify the risk factors that contribute to T2DM development during the postmenopausal years of women with prediabetes.

## Methodology

### Study design

This is a retrospective cohort study including 444 women with a clinical history of impaired fasting glucose (IFG; plasma glucose 100–126 mg/dL) after evaluation in the Endocrinology outpatient clinic at Fundacion Jimenez Diaz Hospital, between January 2013 and December 2018. Among them, 229 individuals were diagnosed as prediabetic after an oral glucose tolerance test (OGTT) in those who repeated IFG and/or showed impaired glucose tolerance (IGT, plasma glucose 140–200 mg/dL after 2-hours glucose overload) or elevation of plasma glycated hemoglobin (HbA1c, 5.7%-6.4%), following criteria of the American Diabetes Association (ADA) (1). Prediabetic women were classified as premenopausal (< 51 years-old) or postmenopausal (≥ 51 years-old) ages according to the Spanish Society of Gynecology and Obstetrics (13). Postmenopausal females were further categorized into early (51–60 years-old) or late (> 60 years-old) postmenopausal stages. All subjects were followed for up to 5.5 years for the diagnosis of type 2 diabetes (T2DM). Exclusion criteria, applied to all women irrespective of age, included pregnancy, uncontrolled hyperthyroidism, menopausal hormone therapy, corticosteroid use, and anti-diabetic treatment. Women with surgically, chemically, or radiation-induced menopause were also excluded. None of the participants were receiving vitamin D supplementation. The study was conducted in accordance with the Declaration of Helsinki and approved by the Ethics Committee of Hospital Fundación Jiménez Díaz (reference 03-2024) on 13/02/2024. Informed consent was obtained from all subjects involved in the study.

### Clinical variables

The body mass index (BMI), family history of diabetes, and presence of hypothyroidism and hypertension were examined. Biochemical parameters such as plasma total cholesterol (TC), triglycerides (TG), low density lipoprotein cholesterol (LDL-C), high-density lipoprotein cholesterol (HDL-C), uric acid, aspartate aminotransferase (AST), and alanine aminotransferase (ALT), gamma-glutamyl transferase (GGT), vitamin D, and creatinine were analyzed. The estimated glomerular filtration rate (eGFR) was also calculated using the CKD−EPI equation, which incorporates serum creatinine, age, and sex to provide a standardized estimate of kidney function. The liver fibrosis risk was evaluated using the fibrosis−4 index (FIB−4), which integrates age, AST, ALT, and platelet count. All variables were obtained from clinical records.

### Statistical methods

Categorical variables were summarized as counts and percentages. Continuous variables were summarized as mean and standard deviation (SD) or median and interquartile range (IQR), as appropriate. Comparisons between groups were achieved using the chi-square test or Fisher’s exact test (categorical variables), or t-test or the Mann–Whitney U test (continuous variables). Associations between risk factors and T2DM occurrence were assessed using Cox regression models with robust standard errors to estimate hazard ratios (HR), 95% confidence intervals, and p-values. The time to T2DM was analyzed using Kaplan-Meier curves and compared with the log-rank test. To evaluate potential collinearity between age and eGFR, we calculated the Pearson correlation coefficient (r) and the derived variance inflation factor (VIF), computed as [1/(1 + r^2^)], where r^2^ denotes the squared Pearson correlation coefficient. A VIF ≥ 2.5 was considered indicative of possible collinearity (14). All analyses were conducted in R (version 4.4.2; R Core Team).

## Results

### Characterization of the prediabetic population

Prediabetic women were 52.7 ± 12.6 years-old, showed a BMI of 30.3 ± 6.73 kg/m^2^, and 48% of them had IFG, 54.6% IGT, and 70.7% exhibited HbA1c ≥ 5.7% (**Table 1**). They also showed hypertension (42.8%) and hypothyroidism (28.4%), and low levels of vitamin D. Interestingly, 97 women (42%) were at premenopausal ages (< 51 years-old) while 132 (58%) at postmenopausal stages (≥ 51 years-old). The prediabetic postmenopausal women were older and exhibited significantly higher presence of elevated HbA1c and hypertension, and increased levels of HDL-C, liver enzymes (AST, GGT), uric acid, creatinine, and the FIB-4 index, but lower of eGFR than those at premenopausal age (**Table 1**).

**Table 1:** Characterization of prediabetic women at pre- and post-menopausal ages.

Variables	Total (n = 229)	Pre-menopausal age (n = 97)	Post-menopausal age (n = 132)	P value
Age years, mean (± SD)	52.7 (12.6)	40.1 (5.75)	61.9 (7.05)	**<0.001**
BMI kg/m^2^, mean (± SD)	30.3 (6.73)	30.6 (7.61)	30.2 (6.03)	0.656
Family history of T2DM, n (%)	78 (34.2)	37 (38.5)	41 (31.1)	0.301
Prediabetic markers				
IFG, n (%)	110 (48.0)	40 (41.2)	70 (53.0)	0.103
IGT, n (%)	125 (54.6)	54 (55.7)	71 (53.8)	0.882
HbA1c ≥ 5.7%, n (%)	162 (70.7)	53 (54.6)	109 (82.6)	**<0.001**
Hypertension, n (%)	98 (42.8)	22 (22.7)	76 (57.6)	**<0.001**
Hypotiroidism, n (%)	65 (28.4)	24 (24.7)	41 (31.1)	0.368
Total Cholesterol, mean mg/dL (± SD)	192 (36.9)	188 (37.5)	195 (36.3)	0.162
LDL-C, mean mg/dL (± SD)	114 (29.7)	111 (30.0)	116 (29.4)	0.234
HDL-C, mean mg/dL (± SD)	54.1 (14.6)	51.7 (15.4)	55.9 (13.7)	**0.030**
Triglycerides, median mg/dL (IQR)	109 (79.0, 161)	105 (77.0, 157)	113 (81.5, 161)	0.441
AST, median mg/dL (IQR)	20.0 (17.0, 24.0)	18.0 (16.0, 24.0)	21.0 (18.0, 25.0)	**0.002**
ALT, median mg/dL (IQR)	19.0 (15.0, 28.0)	18.0 (13.0, 28.0)	20.5 (16.0, 28.0)	0.051
GGT, median mg/dL (IQR)	19.0 (13.0, 30.0)	18.0 (12.0, 27.2)	20.5 (15.0, 31.0)	**0.045**
Uric Acid, median mg/dL (IQR)	4.90 (4.10, 5.80)	4.70 (3.90, 5.50)	5.05 (4.40, 6.00)	**0.012**
Vitamin D, mean nmol/L (± SD)	17.7 (7.77)	17.1 (8.19)	18.2 (7.44)	0.310
Creatin, median mg/dL (IQR)	0.70 (0.60, 0.80)	0.66 (0.60, 0.74)	0.70 (0.68, 0.80)	**<0.001**
eGFR, mean mL/min, mean (± SD)	95.7 (17.4)	108 (13.7)	86.8 (14.2)	**<0.001**
FIB-4, median (IQR)	0.87 (0.67, 1.18)	0.66 (0.53, 0.81)	1.06 (0.86, 1.36)	**<0.001**

Continuous variables are expressed as mean (± standard deviation; SD) or median (interquartile range; IQR), and categorical variables are expressed as percentages (%). ALT, alanine aminotransferase; AST, aspartate aminotransferase; HDL-C, high-density lipoprotein cholesterol; IGT, impaired glucose tolerance; LDL-C, low-density lipoprotein cholesterol; IGT, impaired fasting glucose; eGFR, estimated glomerular filtration rate; and FIB-4, fibrosis-4 index. The bold values indicate p < 0.05.

### Risk factors for T2DM in prediabetic females at pre- or post-menopausal ages

Prediabetic women were followed for 5.5 years for T2DM diagnosis. The incidence of T2DM at premenopausal or postmenopausal ages was 18% and 35%, respectively; respectively. Sixteen cases of T2DM were observed in women aged <51 years, while 51 cases were observed in women aged ≥ 51 years. The time to debut of T2DM was 1.9 and 2.4 years for premenopausal or postmenopausal females, respectively (p= 0.17). Interestingly, women at premenopausal ages who developed T2DM only unveiled higher BMI than those without T2DM (**Table 2**). However, in case of postmenopausal women, those who developed T2DM had higher BMI, prediabetic markers (IFG and IGT), and presence of hypertension than those without T2DM (**Table 2**). They also showed increased plasma uric acid and creatinine, and lower HDL-C, vitamin D, and eGFR.

**Table 2:** Risk factors for T2DM development in prediabetic women.

Variables	Pre-menopausal age			Post-menopausal age		
	T2DM (-) (n = 81)	T2DM (+) (n = 16)	P value	T2DM (-) (n = 98)	T2DM (+) (n = 34)	P value
Age years, mean (± SD)	40.2 (5.32)	39.7 (± 7.77)	0.818	61.7 (± 6.38)	62.5 (± 8.78)	0.617
BMI kg/m^2^, mean (± SD)	29.9 (7.18)	34.2 (± 8.87)	**0.037**	29.4 (± 5.94)	32.3 (± 5.83)	**0.015**
Family history of T2DM, n (%)	29 (36.2)	8 (50.0)	0.453	29 (29.6)	12 (35.3)	0.686
Prediabetic markers						
IFG, n (%)	31 (38.3)	9 (56.2)	0.290	46 (46.9)	24 (70.6)	**0.029**
IGT, n (%)	42 (51.9)	12 (75.0)	0.153	46 (46.9)	25 (73.5)	**0.013**
HbA1c ≥ 5.7%, n (%)	42 (51.9)	11 (68.8)	0.334	78 (79.6)	31 (91.2)	0.203
Hypertension, n (%)	17 (21.0)	5 (31.2)	0.350	48 (49.0)	28 (82.4)	**0.001**
Hypotiroidism, n (%)	21 (25.9)	3 (18.8)	0.754	28 (28.6)	13 (38.2)	0.404
Total Cholesterol, mean mg/dL (± SD)	188 (38.8)	188 (31.2)	0.997	198 (38.0)	186 (29.8)	0.098
LDL-C, mean mg/dL (± SD)	112 (28.9)	110 (36.3)	0.824	119 (29.5)	108 (28.3)	0.079
HDL-C, mean mg/dL (± SD)	51.9 (16.0)	50.4 (12.2)	0.717	57.9 (14.1)	50.0 (10.8)	**0.004**
Triglycerides, median mg/dL (IQR)	102 (75, 157)	125 (88.5, 156)	0.241	110 (76, 148)	136 (94, 171)	0.053
AST, median mg/dL (IQR)	18 (16, 23)	19.5 (16.5, 25.5)	0.404	21 (17.2, 25)	21.5 (18.2, 27.5)	0.270
ALT, median mg/dL (IQR)	17 (13, 27)	20.5 (16.2, 32.5)	0.140	20 (16.2, 27.8)	22 (16, 30)	0.567
GGT, median mg/dL (IQR)	17 (12, 25)	22.5 (18.5, 41.0)	0.068	19.5 (14.2, 31)	25 (16.5, 29)	0.361
Uric Acid, median mg/dL (IQR)	4.6 (3.9, 5.5)	4.9 (3.7, 5.9)	0.553	4.9 (4.1, 5.8)	5.5 (4.7, 6.5)	**0.013**
Vitamin D, mean nmol/L (± SD)	17.2 (7.08)	16.9 (12.7)	0.933	19.2 (7.82)	15.3 (5.38)	**0.002**
Creatin, mean mg/dL (IQR)	0.67 (0.60, 0.75)	0.62 (0.56, 0.70)	0.458	0.70 (0.63, 0.80)	0.80 (0.70, 0.94)	**0.004**
eGFR, mean (± SD)	107 (13.9)	110 (12.7)	0.507	89.4 (11.1)	79.4 (18.9)	**0.006**
FIB-4, median (IQR)	0.67 (0.55, 0.81)	0.57 (0.47, 0.71)	0.121	1.06 (0.86, 1.36)	1.06 (0.81, 1.34)	0.743

At pre- or post-menopausal ages, prediabetic females showed different risk factors for T2DM development. ALT, alanine aminotransferase; AST, aspartate aminotransferase; HDL-C, high-density lipoprotein cholesterol; IGT, impaired glucose tolerance; LDL-C, low-density lipoprotein cholesterol; IGT, impaired fasting glucose; eGFR, estimated glomerular filtration rate; and FIB-4, fibrosis-4 index. The bold values indicate p < 0.05.

As expected, higher BMI was associated with T2DM onset in prediabetic women at both premenopausal [HR (95% CI): 1.08 (1.02-1.15), p = 0.015] and postmenopausal ages [HR (95% CI): 1.06 (1.00-1.12), p = 0.039], even after adjustment for age (not shown). In addition, in postmenopausal women, IGT [HR (95% CI): 2.59 (1.14-5.88), p = 0.023], presence of hypertension [HR (95% CI): 3.91 (1.59-9.59), p = 0.003], and lower levels of HDL−C [HR (95% CI): 0.96 (0.93-0.99), p = 0.009], vitamin D [HR (95% CI): 0.93 (0.88-0.99), p = 0.016], or eGFR [HR (95% CI): 0.97 (0.95-0.99), p = 0.002] remained independently associated with future T2DM even after adjustment for age and BMI (**Figure 1**). Therefore, additional risk factors, at prediabetic stages, may need to be considered in postmenopausal women regarding the development of T2DM. Nevertheless, the postmenopausal period is not a homogeneous physiological stage, and the relevance of each risk factor may vary between early and late post-menopause.

**Figure 1:**
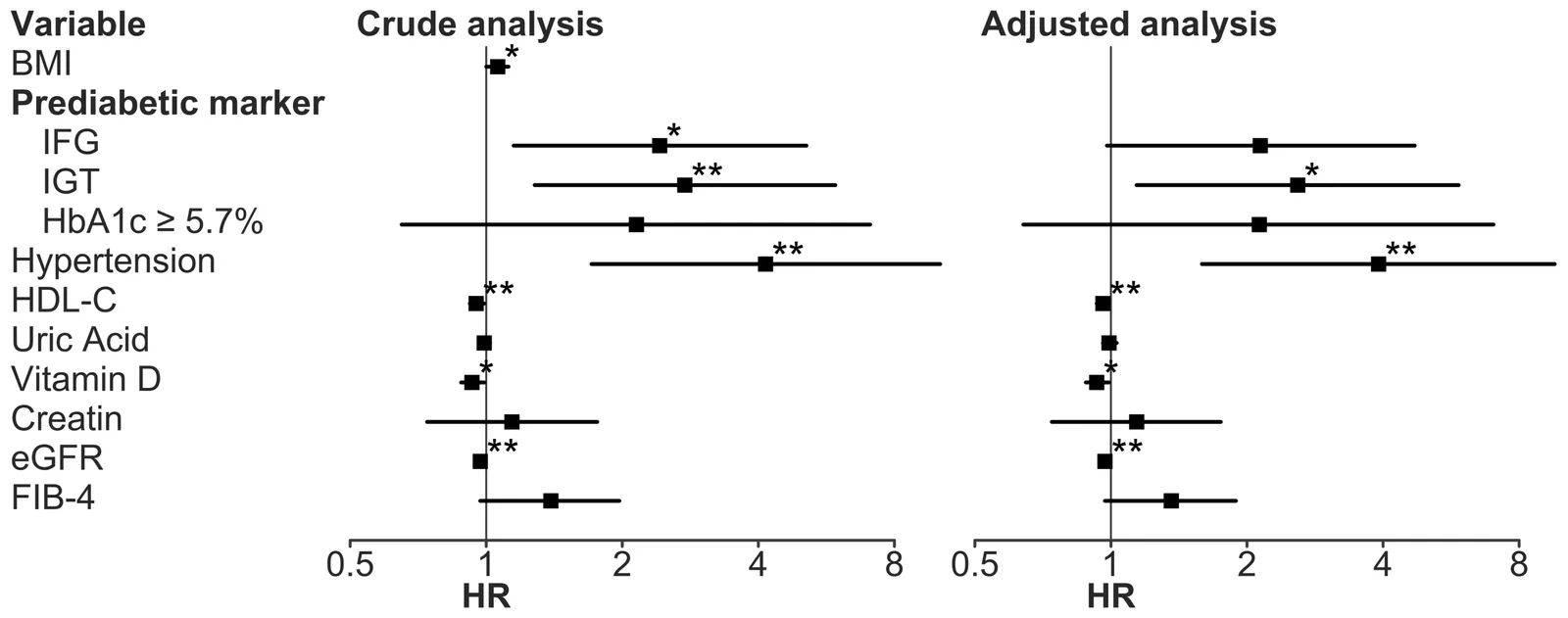
Associations of risk factors with T2DM debut in prediabetic women at post-menopausal age. In the crude analysis, BMI, hypertension, HDL−C, vitamin D, eGFR, and prediabetic markers (IFG and IGT) were associated with T2DM. After adjustment for age and BMI, hypertension, HDL−C, vitamin D, eGFR, and IGT remained significant. HR, hazard ratio. *p< 005 and **p< 0.01.

### T2DM−associated risk factors at early and late postmenopausal stages in prediabetic women

Next, we analyzed the risk factors after stratifying prediabetic women in the postmenopausal stage according to age. Prediabetic women in early post-menopause (51–60 years) showed a higher incidence of T2DM (HR: 2.94, p = 0.007) compared with those in late post-menopause (≥ 60 years), who exhibited a similar incidence than premenopausal women (**Figure 2A**). Thirty-four cases of T2DM were observed in women aged 51–60 years, whereas 17 cases were observed in women aged > 60 years. These findings suggest that early post-menopause may confer distinct risk factors for T2DM occurrence (**Figure 2B**). Indeed, after adjustment for age, increased BMI and uric acid, and reduced vitamin D levels were independently associated with T2DM in early post-menopause (HR: 1.08, p = 0.03; HR: 1.46, p = 0.03; and HR: 0.9, p = 0.024, respectively), whereas presence of hypertension and decreased eGFR was the main determinant of T2DM in late post-menopause (HR: 5.2, p = 0.035, and HR: 0.95, p = 0.002, respectively). Interestingly, Pearson correlation analysis showed that though eGFR was negatively correlated with advancing age (r = -0.67, p < 0.001) and no collinearity was detected between them (variance inflation factor < 2). Together, these findings suggest that the risk profile associated with T2DM progression may shift across postmenopausal stages, from a predominantly adiposity-related metabolic profile in early post-menopause to a more cardiorenal and vascular phenotype in later post-menopause, independently of age.

**Figure 2:**
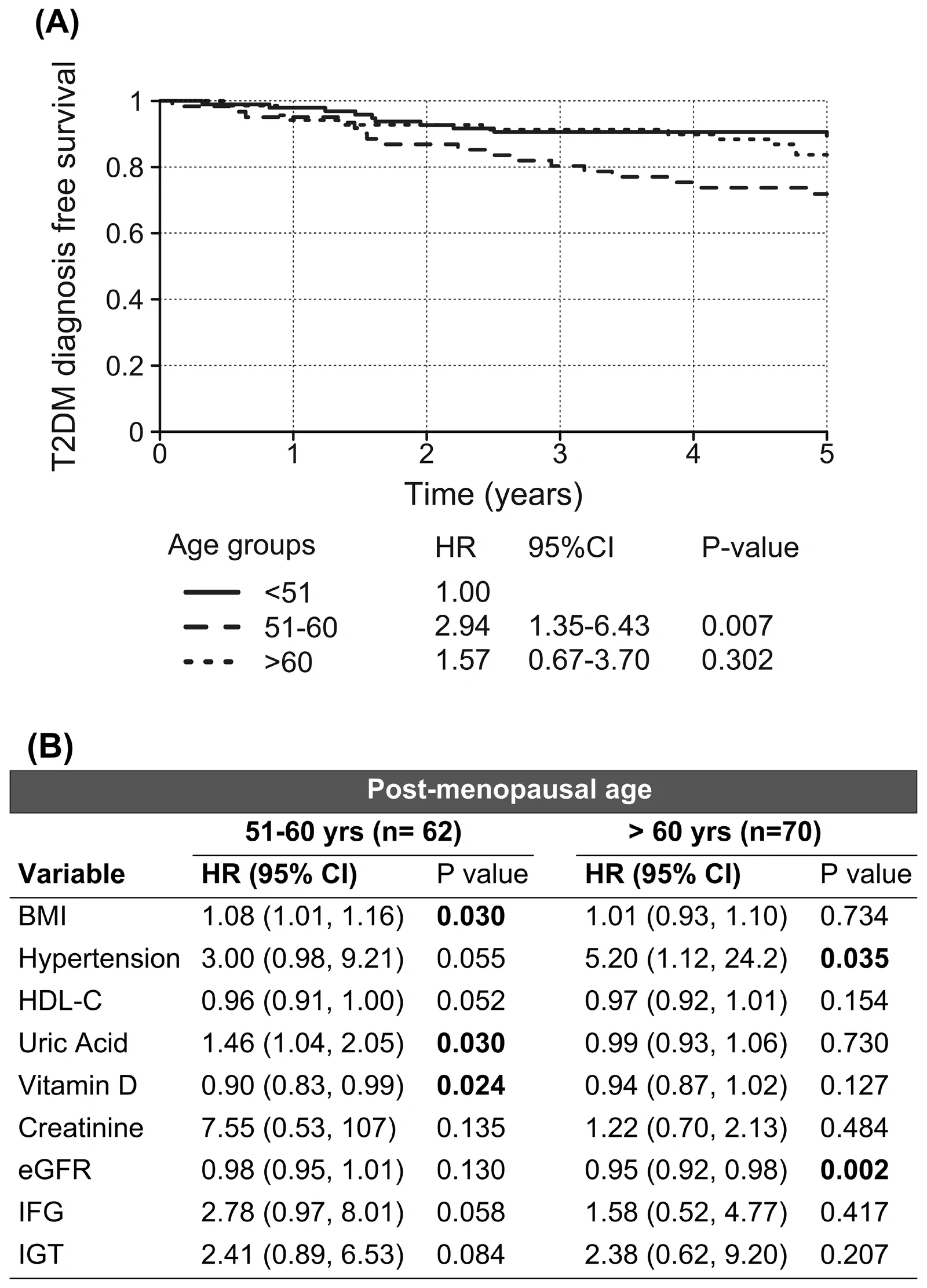
**(A)** Incidence of T2DM in prediabetic women across pre− and post−menopausal stages. The Kaplan-Meier curve depicts the cumulative incidence of T2DM in women younger than 51 years (premenopausal) compared with those aged 51–60 and those older than 60 years (postmenopausal). **(B)** Predictors of T2DM in postmenopausal women. After adjustment for age, higher BMI, increased uric acid, and lower vitamin D were independently associated with T2DM in women aged 51–60 years, whereas presence of hypertension and reduced eGFR were the primary determinants in women older than 60 years.

## Discussion

In this study, we characterized the clinical factors associated with progression from prediabetes to T2DM in women and examined whether these factors differed across age-defined categories used as proxies for premenopausal, early postmenopausal, and late postmenopausal stages. Our findings highlight the substantial heterogeneity within the prediabetic female population and suggest that risk assessment for T2DM may benefit from considering age-related reproductive stage as a clinically relevant framework.

Women with prediabetes showed a substantial burden of metabolic abnormalities, including elevated BMI, hypertension, hypothyroidism, and low vitamin D levels. Compared with premenopausal women, postmenopausal women had a less favorable metabolic profile, with higher HbA1c, liver enzyme levels, uric acid, creatinine, and FIB-4 index, together with lower eGFR (15). These differences are consistent with the metabolic changes associated with estrogen decline, including increased visceral adiposity, insulin resistance, and cardiometabolic dysfunction (10). Accordingly, the five-year incidence of T2DM was higher after menopause, although the time to diagnosis did not differ significantly between groups. When risk factors were analyzed in relation to menopausal stage, BMI was the only independent predictor of T2DM in premenopausal women, suggesting a predominant role of adiposity before menopause (16). By contrast, in postmenopausal women, T2DM onset was independently associated with hypertension, lower HDL-C, vitamin D deficiency, and reduced eGFR, indicating a more complex risk profile after menopause, potentially involving dyslipidemia, renal dysfunction, and chronic low-grade inflammation (17, 18). In this regard, after age stratification, early post-menopause (51–60 years) was associated with the highest incidence of T2DM (HR: 2.94), indicating that the immediate transition to menopause may be critical for metabolic dysregulation. Early post-menopause is characterized by a sharp decline in estrogen levels that amplifies insulin resistance and fat distribution (19), while in late post-menopause (> 60 years), estrogen levels have already stabilized, and aging−related mechanisms may become the predominant drivers of T2DM risk (20, 21). Indeed, in women in the early postmenopausal age group, higher BMI, increased serum uric acid, and lower vitamin D levels were independently associated with progression to T2DM, suggesting that an adiposity-related metabolic profile may be particularly relevant during this phase (22, 23). Elevated uric acid may reflect an adverse metabolic environment characterized by adiposity, low-grade inflammation, insulin resistance, and early vascular dysfunction (24). Moreover, vitamin D insufficiency and hyperuricemia have been reported to coexist in postmenopausal women (25), while Vitamin D deficiency has been linked to impaired beta-cell function and increased insulin resistance (26). The inverse association between vitamin D levels and BMI or visceral adiposity may be explained by sequestration of vitamin D within expanded adipose tissue, altered hepatic metabolism, and chronic low-grade inflammation, mechanisms potentially amplified by menopause-related hormonal and metabolic changes (27). Nevertheless, the role of vitamin D should be interpreted cautiously (28). Because of the retrospective observational design, causality cannot be inferred, and relevant confounders such as physical activity, sun exposure, dietary intake, socioeconomic status, and other lifestyle-related factors were not consistently available. Thus, vitamin D deficiency should be regarded as an associated risk marker rather than a confirmed causal determinant of progression to T2DM.

In older postmenopausal women (>60 years), reduced eGFR and hypertension may reflect an accumulated cardiorenal and vascular burden associated to T2DM development. Estrogen decline has been linked to endothelial dysfunction, increased arterial stiffness, low-grade inflammation, visceral adiposity, insulin resistance, and blood pressure elevation (29). Although renal function declines with both aging and estrogen loss, aging appears to become the dominant driver after 60 years (30). These responses may interact with cumulative vascular damage, contributing to a cardiorenal and vascular risk phenotype. Reduced renal function may favor blood pressure elevation, whereas hypertension may promote renal microvascular damage and progression of chronic kidney disease (31). Also, renal dysfunction may impair insulin clearance, contribute to chronic hyperinsulinemia, alter fasting glucose homeostasis through impaired renal gluconeogenesis, and promote β-cell dysfunction through systemic inflammation and oxidative stress (32, 33). Although the association between eGFR and T2DM progression was statistically significant, its clinical relevance should be interpreted according to the magnitude of kidney failure. Small variations within the normal or mildly reduced range may not necessarily indicate overt renal disease (34). Thus, declining eGFR may represent a marker of broader metabolic and vascular dysfunction associated with T2DM development, independently of age (35). Taken together, these findings suggest that clinical follow-up and preventive strategies in prediabetic women should be tailored according to age-defined menopausal stage. Beyond glycemic control, body-weight management remains a central preventive target. In early postmenopausal women, the association of BMI, vitamin D deficiency, and serum uric acid with T2DM progression supports a broader metabolic assessment, including adiposity-related risk, vitamin D status, and uric acid as a potential marker of metabolic vulnerability. In late post-menopause, the association of reduced eGFR and hypertension highlights the relevance of a cardiorenal and vascular approach, including renal function monitoring, blood pressure control, and strategies aimed at preserving kidney and vascular health. In addition, progression from prediabetes to T2DM is likely driven by a complex interaction of clinical, metabolic, inflammatory, and biological susceptibility factors. Previous studies have suggested that inflammatory and genetic pathways, including *ICAM1* rs5498 polymorphism and PTP1B-related mechanisms, may influence diabetes susceptibility and metabolic regulation (36, 37).

Although menopause has been widely studied in relation to cardiometabolic risk and incident T2DM in broad populations (38), fewer studies have focused on progression from prediabetes to T2DM within postmenopausal subgroups. Importantly, our study does not redefine the clinical diagnosis of menopause, which remains retrospectively established after 12 consecutive months of amenorrhea in the absence of other causes (39). Because menstrual history, age at natural menopause, and hormonal data were not consistently available due to the retrospective design, age-based groups were used as pragmatic proxies for menopausal stages. Therefore, our findings should be interpreted as hypothesis-generating differences across age-defined menopausal-stage groups rather than biologically confirmed menopausal categories. This approach may have introduced non-differential misclassification, particularly around the menopausal transition, potentially attenuating the observed associations. Nevertheless, our results suggest that women with prediabetes may exhibit distinct risk profiles across the postmenopausal years, supporting risk assessment based not only on postmenopausal status but also on relevant metabolic, vascular, and organ-function markers. Future prospective studies including detailed reproductive history, hormonal assessment, and comprehensive evaluation of potential confounders are needed to validate these findings.

## Limitations

Several additional limitations should be acknowledged. This was an observational, single-center study based on retrospective medical records, which may limit the external validity of the findings. In addition, only women with complete clinical histories were included. While this criterion ensured greater consistency and reliability of the analyses, it may also have introduced selection bias. Moreover, several relevant lifestyle and psychosocial factors, such as dietary patterns, physical activity, sleep disturbances, and depressive symptoms, were not systematically recorded. These factors are common in this population and may influence adiposity, vitamin D status, blood pressure, renal function, uric acid levels, and glucose metabolism. Therefore, residual confounding cannot be ruled out, and the results should be interpreted as hypothesis-generating.

## Conclusion

The risk of progression from prediabetes to T2DM in women may differ across age-defined menopausal stages, with distinct clinical risk profiles emerging over time. Excess body weight appears to be a key factor before menopause, whereas early post-menopause may be characterized by a broader adiposity-related metabolic profile involving BMI, vitamin D deficiency, and increased uric acid. In late post-menopause, reduced eGFR and hypertension suggest a shift toward a cardiorenal and vascular risk phenotype. These findings should be interpreted cautiously given the observational design and potential residual confounding, but they support the value of stage-specific follow-up and personalized prevention strategies in women with prediabetes.

## Data Availability

The original contributions presented in the study are included in the article/supplementary material. Further inquiries can be directed to the corresponding author.
